# Advancing equity and justice through community science programming in design, construction, and research of a nature-based solution: the Duwamish Floating Wetlands Project

**DOI:** 10.1007/s42532-022-00123-9

**Published:** 2022-10-20

**Authors:** Leann Andrews, Ashley D. Mocorro Powell, Nancy Rottle, Jennifer Engelke

**Affiliations:** 1 Department of Landscape Architecture, The Pennsylvania State University, State College, PA USA; 2 Green Futures Research and Design Lab, University of Washington, Seattle, WA USA; 3 Department of Landscape Architecture, University of Washington, Seattle, WA USA; 4 Green Futures Research and Design Lab, College of Built Environments, University of Washington, Seattle, WA USA

**Keywords:** Citizen science, Community science, Floating wetlands, Environmental justice, Nature-based solutions, Ecological restoration

## Abstract

Dx^w^dəw refers to the Black-Green Rivers confluences that made the Duwamish River in Seattle, Washington, USA, prior to the 1910s. Significant industrial activity and human-made diversions to these rivers caused heavy pollution and eliminated 97% of historic wetlands, forever altering the historic river systems, salmon runs and human and aquatic health. Today the Green-Duwamish River and Duwamish Estuary are an industrial and commercial corridor, albeit also a site of cultural significance and fishing rights for urban Indigenous and Coast Salish tribes, and home and workplace to diverse urban populations of sustenance fishers, immigrants and refugees, communities of color, and low-income neighborhoods. Using a socio-ecological and environmental justice perspective within a nature-based solution, the Duwamish Floating Wetlands Project designed and piloted four constructed floating wetland structures for two years on the Duwamish River and researched their feasibility to provide habitat for out-migrating juvenile salmon. A multi-pronged community team (community leaders, liaisons, stewards and scientists) worked alongside academics and professionals. This paper showcases the formulation and adaptation of a two-year citizen/community science program integrated into the project. We outline the frameworks, approach, outcomes, and lessons-learned of the community science and outreach program, and compiled these in a list of guidelines to provide practitioner, researcher and community insight into the value and necessity of prioritizing environmental justice, racial equity, and ecosystem needs in nature-based solutions.

## Introduction

### Nature-based solutions and social equity

Nature-Based Solutions (NBS) can be defined as urban ecosystem-inspired designs that address local to global intertwined socio-ecological issues and are best implemented and assessed through close collaboration with communities, stakeholders and professionals (IUCN 2022; Bayulken et al. 2021). Effective NBS projects are crafted to address specific socio-ecological problems as defined by locals, and as such, often require a flexible, adaptive and inclusive approach within their design, assessment, and management (La Rosa et al. 2021, p. 331). Despite the potential for NBS to simultaneously strengthen social and ecological systems, there are limited NBS projects to date that place social equity and justice at the forefront of their ecological goals, compelling researchers to call for “*just* nature-based solutions” (Cousins 2021), and reflections on deeper forms of citizen participation and equitable inclusion within NBS (Kiss et al. 2022, p. 12). This article within a special issue on “socio-ecological perspectives on NBS” showcases a community science program within an NBS that began with goals of environmental justice and social equity, and embeds community health and opportunity alongside ecosystem health. We share lessons learned and provide guidelines for future teams interested in working with community scientists.

## Project background

### Context of people and place

**Fig. 1:**
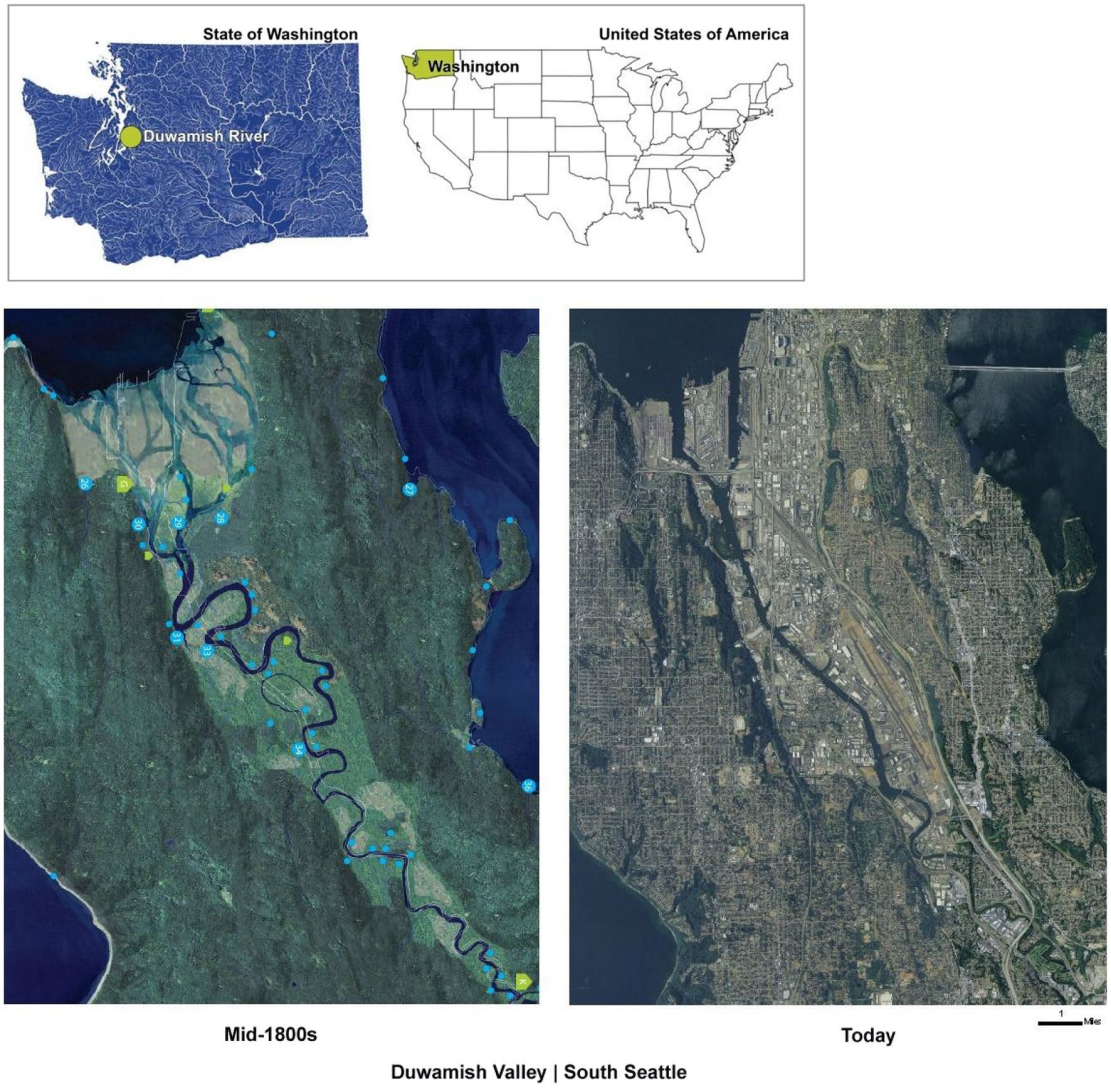
The Duwamish River is located in the State of Washington in the USA. The industrialization of the Duwamish River, in Seattle, USA, has greatly altered the river and wetland ecosystems, negatively impacting human and ecological health [image credit: modified from the Waterlines Project, publicly available online at the Burke Museum, https://www.burkemuseum.org/collections-and-research/culture/archaeology/waterlines-project]

In the Pacific Northwest of the USA, salmon are a unique migratory keystone species that live in freshwater rivers or lakes as juveniles, journey out to the saltwater sea for several years, and then return to the rivers to reproduce and die. The Duwamish River in Seattle, Washington, is the ancestral home of the Dxʷdəwʔabš (Duwamish Tribe) and other Coast Salish Peoples. Through the Treaty of Point Elliott (1855) and Boldt (1974), treaty tribes retained sovereignty to fishing, hunting, and gathering rights. However, significant urban-industrial activity and human-made diversions to the Green-Duwamish river altered its historic ecosystems and greatly diminished salmon populations, with 97% of wetlands replaced with shoreline armored for commercial and industrial uses. See Fig. 1. With significant legacy and ongoing pollution, 5.5 miles and 412 acres of the lower river estuary were designated as a Superfund Site (meaning severe pollution prioritized federal funding towards cleanup efforts), Chinook salmon are on the Threatened Species list, salmon-eating Southern Resident Orca Whales are on the Endangered Species list, and other river fish and crustaceans are no longer safe to eat, impacting the fishing rights and deep cultural connections of Indigenous peoples to salmon, orcas and the river (Duwamish Tribe 2022).

The Duwamish River valley is also home to two of the lowest income and most diverse neighborhoods in Seattle, with 42% foreign born and 70% non-white (U.S. Census 2013). While culturally rich, these communities were historically disinvested from discriminatory housing policies, industrial neighbors, and a highly polluted and unhealthy river (University of Washington 2022). All these factors contribute to significant impacts on community health and wellbeing; Duwamish residents live on average eight years less than the typical Seattleite, and 13 years less than affluent white Seattle neighborhoods (Gould and Cummings 2013, p. 38). The project discussed in this article acknowledges close connections that multiple peoples and their histories have to the Duwamish River and sought to address these environmental injustices by making communities an integral part of NBS and salmon restoration efforts.

### Floating wetlands as nature-based solutions

**Fig. 2:**
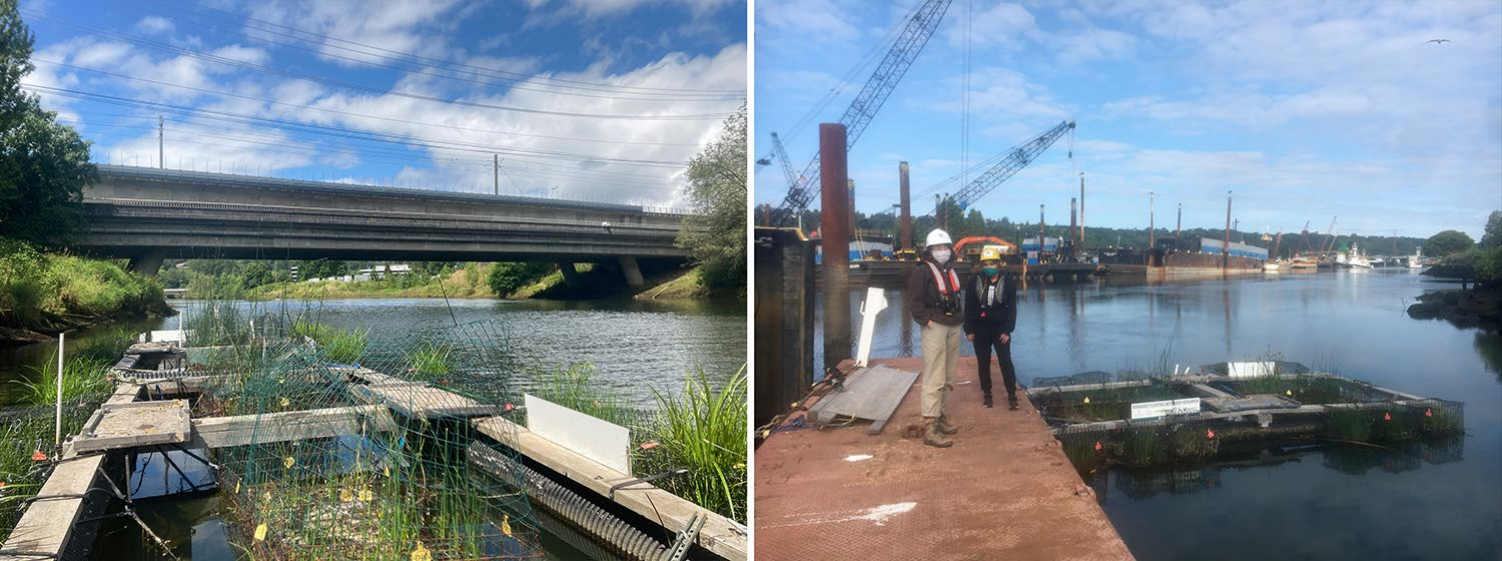
Images from June 2022 (left) and April 2022 (right) showing the floating wetland “Biobarges” (the larger monitoring structure) with floating wetland “Biofilters” and monitoring equipment attached, deployed on different sections of the Duwamish River Estuary, and pictured with Community Scientists. [Image credit: the Duwamish Floating Wetlands Project Team, UW Green Futures Lab]

Constructed floating wetlands can be implemented as an NBS to provide creative solutions to river restoration in urban and industrial areas having little space for traditional land-based restoration. Floating wetlands can improve water quality, provide aquatic habitat, lower algae biomass and improve biodiversity (Bi et al 2019). Integrating public outreach and/or community engagement within floating wetland projects may increase the sustainability and public acceptance of floating wetlands (Jinadasa et al. 2019; Ware 2019, pp. 8–10), therefore providing mutual social and ecological benefits. The Duwamish Floating Wetlands Project piloted four constructed floating wetland “Biobarges,” each containing four “Biofilters,” on the Duwamish River and tested the capacity of these NBS to provide vital habitat for out-migrating juvenile salmon in this urban-industrial river. See Fig. 2 and our project website https://livingshorelines.be.uw.edu/. Prototypes were constructed and assessed over two years (2019 and 2020), and the project took a safe-to-fail adaptive management approach, reacting to community and stakeholder input and reflecting upon lessons learned from the first year to make changes in the second year (Rottle et al 2022). Community engagement and outreach was embedded within each project stage.

### Framing the Duwamish floating wetlands community science and outreach program

Academic-led research and ecological restoration projects may unintentionally cause harm to communities experiencing environmental injustices with power dynamics and academic requirements creating barriers to community-driven efforts and degrading trust with “in and out” self-serving timelines (Shrader-Frechette 2017; Davis and Ramirez-Andreotta 2021, p. 026001-2). In addition, the STEAM (science, technology, engineering, arts, mathematics) fields are experiencing a “leaky pipeline,” losing students at every stage of the education pathway from middle school to higher education. Minority, low-income, and female students are particularly vulnerable to the STEAM “leaky pipeline,” and linked to histories of racial redlining impacting public school funding (NCSES 2019). This lack of representation is particularly detrimental to communities experiencing environmental injustices who need STEAM professionals who can relate to their struggles to join their advocacy.

Because of the aforementioned environmental injustices in the Duwamish Valley, it was vital that community engagement be an integral component of the Duwamish Floating Wetlands Project, and crafted in a meaningful and inclusive way to stimulate real change in the local and scientific communities. Citizen Science is one method of engaging community members. Citizen Science (and its sub-terms community-engaged research CEnR, community-based participatory research CBPR, and community-owned and managed research COMR) engages the public within research and promotes education and empowerment with an eye towards policy and systemic changes, going beyond conventional publication-driven research agendas (Cooper et al. 2021). Because of the large number of undocumented and Indigenous peoples that may not identify with the word “citizen,” and the drive to diversify from white, educated, above middle-income participants often found in citizen science, the Duwamish Floating Wetlands Project substituted the terminology “community science.” While the team is still reflecting upon the (positive or negative) impact the term community science may have (Cooper et al. 2021), our goal was action-oriented research with the broadest community participation we could conduct in the constraints of a pilot restoration project.

The Community Science and Outreach Program for the Duwamish Floating Wetlands Project was grounded in several conceptual frameworks and best practices aimed at increasing opportunity for those the furthest from justice. These included: Culturally Sustaining Pedagogies (Paris and Alim 2017), Principles of Environmental Justice (Principles of Environmental Justice 1991), the Rose Foundation Theory of Change (The Rose Foundation 2018), Place-based Education (Smith 2017), and Youth Participatory Action Research (Ozer et al. 2020).

## Project approach

### Goals

Because of its “safe-to-fail” adaptive approach to maximize the sustainability of the NBS (Ahern 2011), the first year of the Duwamish Floating Wetlands Project piloted the Biobarge and Biofilter prototypes, the research methods for measuring their impact on creating habitat for outmigrating juvenile salmon, and the community science and outreach program. This “proof-of-concept” developed in the first year was then reapplied with adaptations in a second year of the study (Rottle et al 2022).

The overall goal of the community science and outreach program was to infuse equity and access at the forefront of training and monitoring of this NBS. After talking with community groups, ecologists and authorities, the specific goals of the community science program for the first pilot year were determined: (1) to engage a wide diversity of Community Scientists in the field research, considering age, gender, race and ethnicity, geographic location, education level, income, and experience level; (2) to prioritize youth and emerging scientists to foster early STEAM learning and educational retention; (3) to inform, educate and inspire the general public in Seattle to build public momentum in support of urban ecological restoration using NBS; and (4) remain mindful of project capacity and realistic commitments of a pilot program while finding a way to engage all who expressed interest. Networking with community groups and individuals during the first pilot year then led to additional goals for the second year of the study: (5) target engagement with residents of the Duwamish Valley; and (6) with the onset of the COVID-19 pandemic, adapt the program to provide remote and socially distanced opportunities.

Before its launch, the team conducted significant outreach to organizations working in the Duwamish Valley to ensure project goals aligned with efforts already on the ground and to help spread the word about opportunities for community science involvement. This outreach was conducted over two months each year, via primarily in-person visits to gain trust and prioritize participation of historically underrepresented communities. The team connected with two dozen community organizations who guided project framing, recruitment and public engagement.

### The team

The initial project team, consisting of three white and Western university-trained professionals, recognized early in project development that a dedicated Community Science and Outreach Lead & Strategist (hereon referred to as Community Science Lead) was needed to elevate the community contributions in the project. Each year, the project benefited from the Community Science Lead’s identities reflecting the demographics and experiences of the surrounding area and community groups, and their personal connections to these communities. The first year was a self-identified Black woman who was studying public administration at a public university and had a background in environmental science, and the second year was a self-identified first-generation scientist, low-income earner, and person of color with lived experiences in the Green-Duwamish Valley and Washington public school systems. Both individuals held intersecting identities, intimate lived experiences as women of color in the field, and STEAM academic experiences that overlapped with the team’s academic scientists.

Community Science Leads served as bridges between the project leadership team, academic scientists and local community members (i.e. Community Scientists) and community organizations. During their corresponding years, these Community Science Leads joined the Community Scientists out in the field to increase relational continuity, confirm safety and best practices, guide consistent measurements, and ensure clear communications were shared with local community-based organizations. Community Science Leads also organized opportunities to share the work with broader public audiences. In the second year, the team learned the potential of expanding project presence through established community social media networks, and hired two specialists to increase online communication efforts. In addition, with a more dedicated focus towards recruiting community members from the local neighborhoods in the second year, a Community Liaison was hired. This Community Liaison was a bilingual person of color, resident of the local neighborhood, immigrant, trained in engineering, and a community activist for the local, urban immigrant and refugee sustenance fishers and Latine/x communities who frequently engage in cultural practices on the Duwamish River.

### Funding

The community science program comprised about 20% of the total project budget with funds coming from a private foundation, public university grant, and the Port of Seattle. All team members, including Community Scientists, were offered compensation for their time and expertise, either in fair wage monetary payment or, for those enrolled in a high school International Baccalaureate program (IB) or university and who expressed the need, the option for service-learning hours or academic credit. All forms of compensation and credit were fundamentally critical to achieving the equity and access goals of the project with best practices to ensure active participation from households or learners who may be asset-limited or income-constrained (Washington Poverty Reduction Work Group 2018, p. 14). It is important to note that the Community Science Leads, Community Liaison, and academic NBS designers and scientists were also an extension of these equity and access goals. It was critical that the project had funds to compensate the Community Science Leads, Community Liaison and communications staff for their transformational leadership and strategies to see mutual impact on this project, and Community Scientists for their time, effort and invaluable lived knowledge.

### Community scientist involvement

**Fig. 3:**
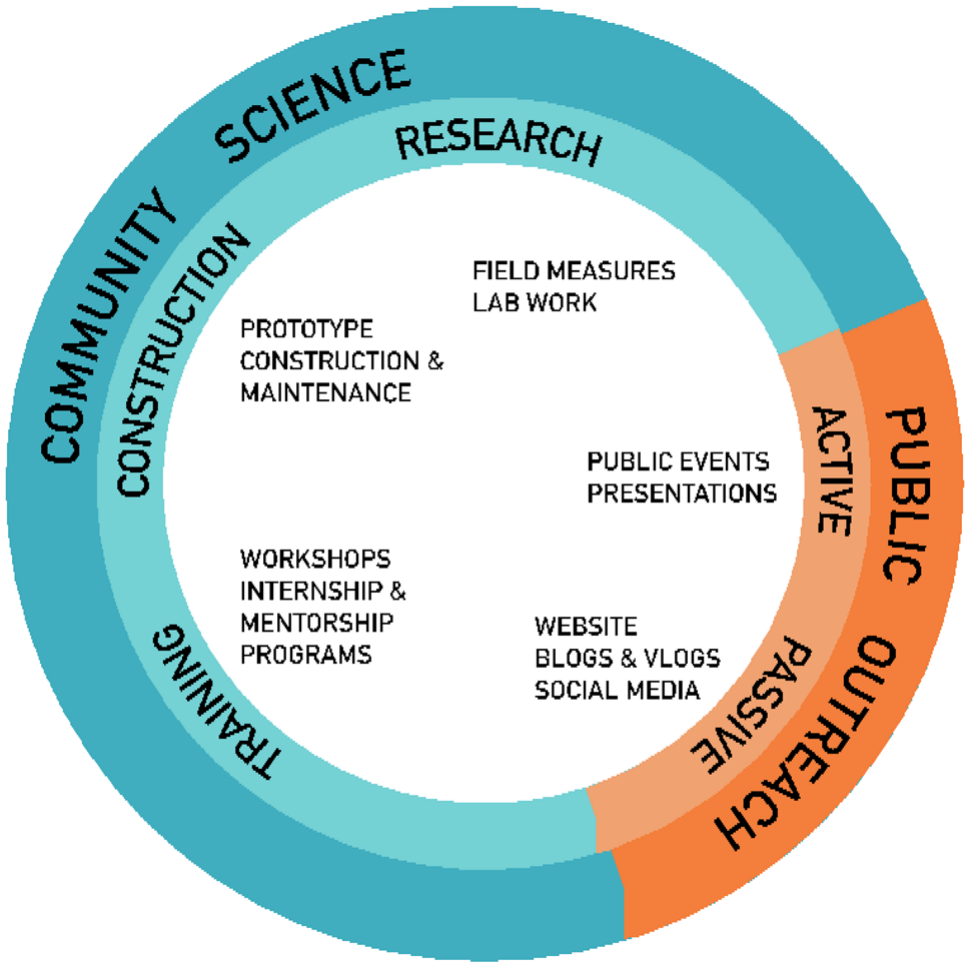
Community engagement strategy for the Duwamish Floating Wetlands Project. Community engagement consisted of a community science program incorporating community members within construction, research and training of the floating wetlands, and public outreach took both an active and passive strategic approach. [Graphic credit: the Duwamish Floating Wetlands Project Team, UW Green Futures Lab]

The Community Science Leads and Community Liaison worked with project leadership to establish a strategy of different intensities of community engagement and public outreach opportunities. See Fig. 3. Over two years, Community Scientists were engaged in prototype construction and maintenance, field measures, lab work, and both on-site and remote workshops. The project hosted an internship program for undergraduate scholars, and in the second year, a mentorship program for interested youth and students.

#### Community in prototype construction and maintenance

**Fig. 4:**
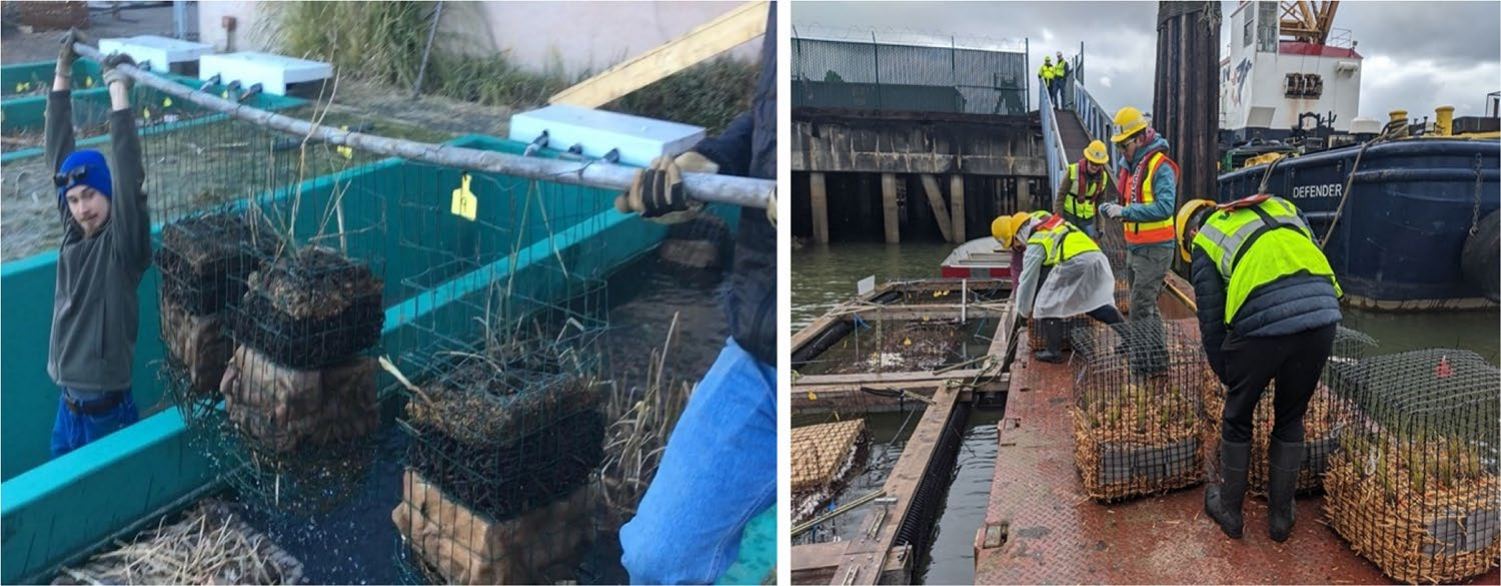
Construction of prototypes for the Duwamish Floating Wetlands Project. Images show students constructing floating wetland “Biofilters” at an underutilized hatchery in February 2019 (left) and community members and the academic team installing the Biofilters into the Biobarges at a dock on the Duwamish River in March 2020 (right). [Image credit: The Duwamish Floating Wetlands Team, UW Green Futures Lab]

Building from designs produced in a university floating wetlands seminar, an interdisciplinary group of university students and community members worked with project leadership to construct the first Biofilter prototype. After analyzing first year research results, project leaders led a university class in the second year to construct another Biofilter prototype to add onto the Biobarge frame. Community members then joined a series of work parties to repair the initial Biofilters and install the new Biofilter prototypes. Four community members who lived next to the Biobarges, including the Community Liaison, provided maintenance in this second year, with guidance from project leadership and coordination with the Community Science Lead. This was especially critical during the stay-at-home mandates and supported the greatly reduced transit options available for the academic team during the early months of the COVID-19 pandemic. See Fig. 4 for construction images.

#### Community in field measures

One to three Community Scientists joined the Community Science Lead and/or academic team on the river each field shift to observe fish, measure plants, collect invertebrates, take water quality measures, and document photos and field notes. Measures were staggered in the day and week so one measure did not disturb another, which also accommodated more community scientists. In year one, Community Scientists accessed the Biobarges by boat and standing directly on the floating Biobarges, whereas in year two the pandemic shutdowns required the team to dock the floating wetlands since boat access was not possible due to pandemic restrictions. In addition to the advantage of adequate water depth during low tides at the dock sites, accessing the floating wetlands via a dock also increased accessibility and comfort for Community Scientists, a lesson learned from first year feedback. Community Scientists could make choices about participating in field activities matching their personal comfort around water and could either take measures from the dock or boat, or gradually build confidence to stand on the floating wetland structures.

Community Scientists were trained with research protocols each time they went out into the field. Research consistency was maintained in the field with presence of the Community Science Lead and academic scientists. The Community Science Lead also trained the academic scientists on how to mindfully and meaningfully incorporate Community Scientists in field research activities. Both academic and community scientists were trained on field health and safety protocols in the urban industrial and estuarine contexts, signed waivers and university forms, and were provided with field and risk management gear (e.g. hard hats, reflective vests, life vests, water safety throwline, gloves, sunglasses, clipboards etc.), transportation if needed, and (in the second year) COVID-19 supplies (e.g. masks, cleaning supplies, hand sanitizer etc.) for the project.

Community Scientists collected data to determine the impact the floating wetlands had on providing habitat for out-migrating juvenile salmon. For fish measures, Community Scientists helped count and identify juvenile salmon near the Biobarges and at reference and control sites. They also set up and collected GoPro cameras that captured underwater footage of fish at the Biobarges. For plant measures, Community Scientists determined the percent plant cover, plant height, and mortality rates. For invertebrate collection, Community Scientists set out fall traps on the Biobarge, reference, and control sites to collect terrestrial invertebrates that fell from the plants; 24-h later Community Scientists collected the containers and strained the invertebrates into jars for lab analysis. In the second year Community Scientists also collected aquatic invertebrates from the substrate. For water quality measures in the first year, Community Scientists were trained in YSI EXO2 sonde protocols and collected data with the academic scientist. At the end of the project, Community Scientists helped collect plant specimens and soil substrates for metal, biomass and nutrient testing. Finally, Community Scientists used a project tablet computer to take photographs of each Biofilter to systematically document design durability and plant growth.

**Fig. 5:**
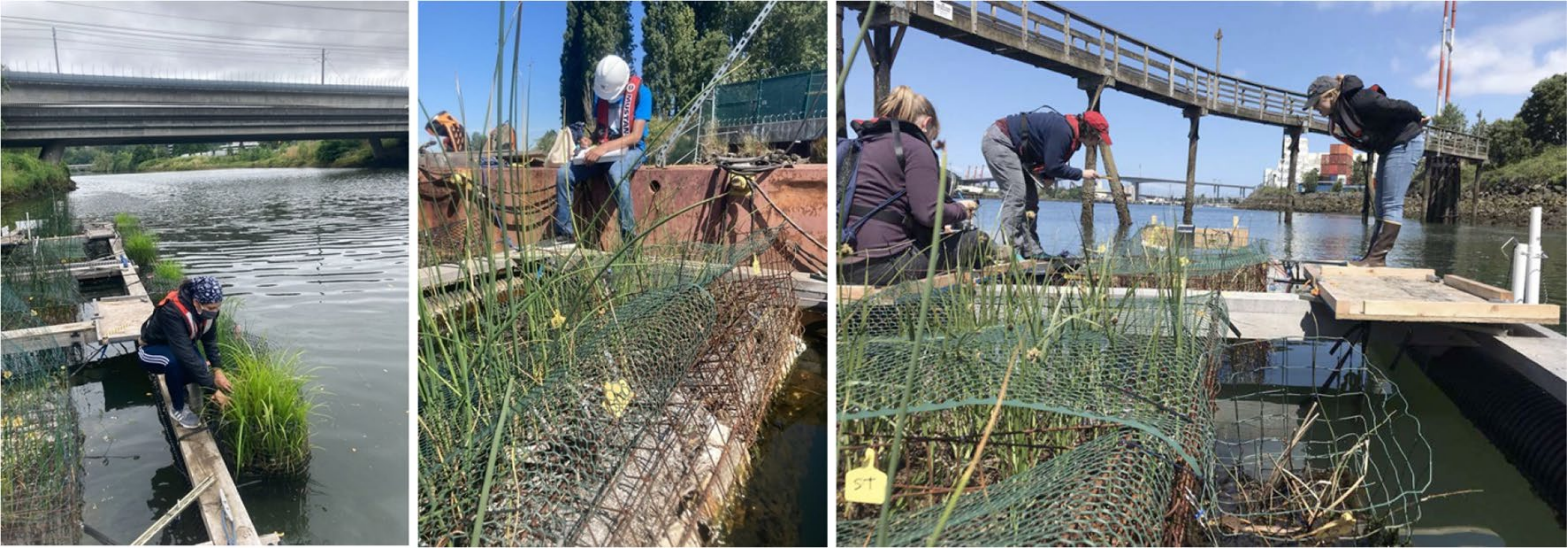
Community Scientists and the academic team taking field measures for the Duwamish Floating Wetlands Project in June 2020 (left), May 2020 (middle), and June 2019 (right). [Image credits: The Duwamish Floating Wetlands Team, UW Green Futures Lab]

Field research occurred during peak out-migrating salmon season, approximately April through July, with each measure taken once per week. The Community Science Lead crafted the field schedule to prioritize Community Scientists’ personal interests (example: wanting to see fish) and preferred schedules. During field monitoring shifts, Community Scientists had the opportunity to rotate between stations (e.g. plant station to fish station) to break up field activities. These rotations enhanced time with multiple team members, increased individual attention spans, and built enough confidence over multiple shifts to co-lead an activity if they so chose. See Fig. 5.

#### Community in lab work

**Fig. 6:**
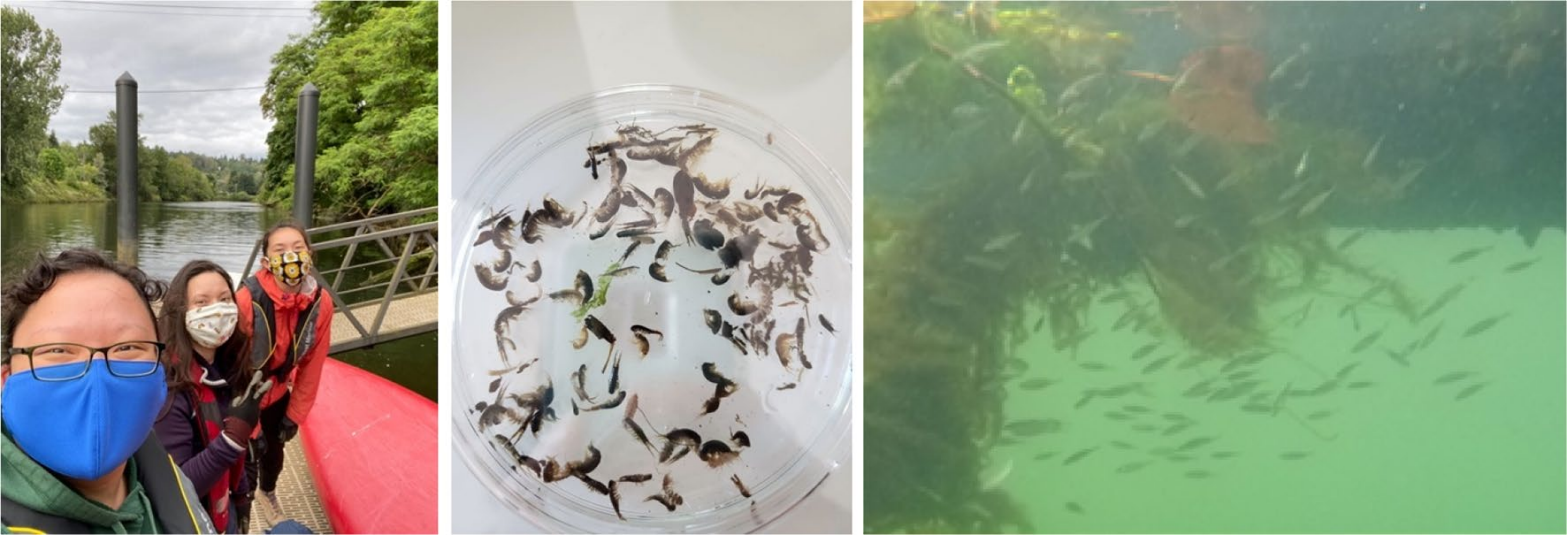
Community Scientists and the Community Science Lead engaging in field-to-lab work analyzing invertebrates and juvenile salmon for the Duwamish Floating Wetlands Project in June 2020. [Image credits: The Duwamish Floating Wetlands Team, UW Green Futures Lab]

Community Scientists joined academic scientists at a university lab to categorize and count invertebrates gathered in the field. Community Scientists worked with academic scientists to analyze the collected invertebrate samples under a microscope, and then completed a spreadsheet with information on when the sample was collected, where it was collected from, the Taxa observed and the count of invertebrates. Trained academic scientists were present to help answer questions and work through the process with them. In the second year, Community Scientists also participated in remote data analysis activities, analyzing hundreds of hours of underwater fish GoPro video data to identify and quantify fish. Lab work was intensive and required great attention to detail and so was reserved for Community Scientists that needed lab experience or academic credit for their studies. See Fig. 6.

#### Community in workshops

**Fig. 7:**
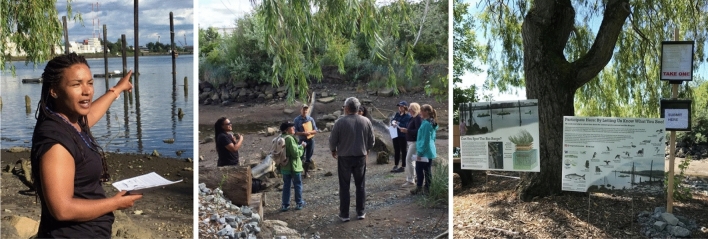
On-site workshops orchestrated by the Community Science Lead during the first year of the Duwamish Floating Wetlands Project in May 2019. [Image credits: The Duwamish Floating Wetlands team, UW Green Futures Lab]

In year one, the Community Science Lead invited community members with scheduling conflicts to join a half day on-site workshop to explain the floating wetland systems, monitoring procedures, and provide training for documenting bird and aquatic mammal observations from the riverbank. See Fig. 7.

#### Community in internship and mentorship programs

In both study years, two partner organizations hosted undergraduate interns from demographic groups underrepresented in the environmental and conservation sectors. The interns were supervised by the Community Science Lead and engaged in field, lab and communications activities to contribute to the larger research project while exploring their own interests to advance their careers. These emerging scientists created short independent projects in an area of interest which built upon the Duwamish Floating Wetlands work. With stay-at-home mandates at the beginning months of the pandemic, alternative modes of engaging community members were explored. The Community Science Lead organized online bi-weekly mentorship meetings with interested youth and emerging scientists in high school and early undergraduate studies. Mentorship participants could talk with senior team members, learn about diverse careers in design and science fields from professional guests, and discuss their individual future plans.

### Public outreach and education

In addition to the involvement of Community Scientists, the Community Science Lead developed a broad public outreach strategy that included both active and passive approaches.

#### Active public outreach

**Fig. 8:**
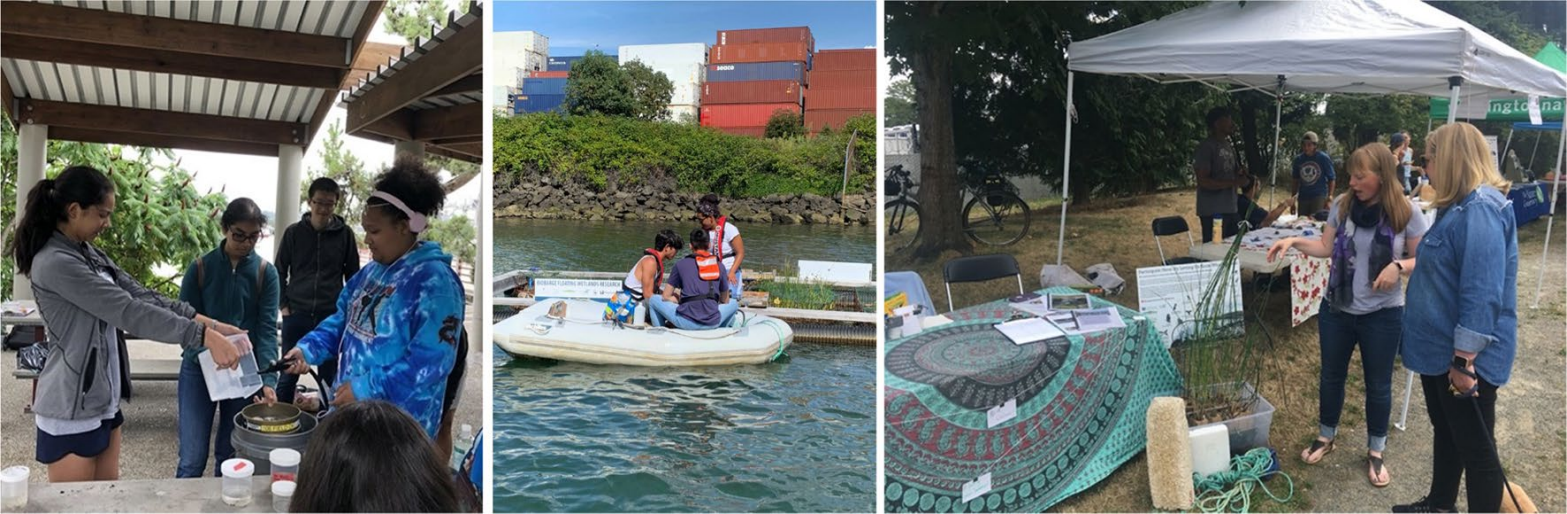
Public outreach events in 2019 with hands-on demonstrations for the Duwamish Floating Wetlands Project. Images show a youth summer camp (left), a guided tour with the Doris Duke Conservation Scholars (middle), and an interactive display at the Duwamish River Festival (right). [Image credits: The Duwamish Floating Wetlands Team, UW Green Futures Lab]

The Community Science Lead organized community events, activities and presentations that shared the project with local communities and those who might not otherwise have access to the project. During the first year, public outreach included prototype demonstrations at the Duwamish River Festival, a summer youth program with the Pacific Science Center, a tour with the Doris Duke Conservation Scholars (DDCSP), a workshop with the Sustainability in Prisons Project at the Washington Corrections Center for Women, a panel presentation at the Salish Sea Environmental Justice Panel, and presentations at several classes at the local public university. While the same level of active public outreach was planned for the second year, in-person activities were canceled due to the onset of the COVID-19 pandemic in 2020. Virtual presentations occurred at the Green-Duwamish Watershed Symposium, Young Women Empowered STEM Exploration Day, DDCSP, Seattle MESA Summer Program, and YWCA Seattle’s Femme2STEM program. Additionally, team members were interviewed by the local TV station and for a popular science podcast. See Fig. 8.

#### Passive public outreach

In the first year, the team established a social media account, and in the second year expanded the passive public outreach by developing a public-facing website for the project, content for blog and video blogs (vlogs), and weekly social media postings to hold an active digital presence. This expansion was in part due to efforts to redirect public engagement that was not possible in-person during the early months of the COVID-19 pandemic, and in part to stay connected with the community networks established in the first year of the study. Online content could be “shared” with these organizations and community members to post on their social media and more effectively spread the word to local community members. This was a particularly helpful engagement method for community members who spoke different languages than English, as social media platforms facilitated automatic translation and captioning of text and video. In addition to the online public outreach, in the first year the team installed graphic signage along the bank of the river to invite education about the floating wetland systems to the general public.

### Community science in a global pandemic

The onset of the COVID-19 pandemic presented significant challenges for field and lab work in the second year of the study, which began in March 2020. The project implemented strict health and safety protocols and social distancing requirements that were updated every few weeks as the world learned more about the virus. The project budget shifted to include cleaning products, personal protection equipment, increased personnel hours, and increased single- user vehicle use. With closure of the staffed university lab, the project purchased microscopes and set up a makeshift invertebrate lab in an unused classroom on campus. When Seattle implemented “stay-at-home” mandates, the community science program adapted. Onboarding, training and community outreach was shifted to hybrid and online formats. Research methods increased remote monitoring equipment such as underwater GoPro cameras to capture fish data. For the first eight weeks of field monitoring, the research team harnessed the capacity of four enthusiastic community members who lived near the floating wetlands—a family “pod” who could safely monitor two of the floating wetland units near their house, and one resident who hosted two of the floating wetland units at his private residential dock and could perform work while still “staying-at-home.” These residents embraced the paid leadership opportunity, performing twice weekly maintenance, collecting data from remote monitoring equipment, and documenting fish and plants. They were critical to continuance of the research, as the academic scientists were unable to go to the field for the first six weeks due to university and regional work restrictions, while the out-migrating juvenile salmon season would not support project delays. By June, when requirements began to lift, the Community Science Lead organized a blend of in-person and online opportunities for engagement (socially distanced field monitoring, analyzing GoPro footage, an online mentorship program, remote workshops and social media posts). These opportunities that emerged from significant obstacles ended up becoming assets to the program: the project became more accessible to community members who did not feel safe in the field (COVID-19 or other health concerns); it expanded the reach of the community science programming to those residing outside of Seattle; and provided welcome financial assistance and time outside in a community setting during an isolated and trying time for all team members.

## Outcomes

### Documentation of community science involvement

Because community involvement requires an adaptive and flexible approach, measuring success in traditional scientific ways may be difficult or even inappropriate in social aspects of NBS (La Rosa et al. 2021). After much team discussion, ultimately it was determined that implementing a survey to measure community successes would be detrimental to the spirit of the program; it would make the community members (many of which came from vulnerable groups) the subjects of study and might reinforce feelings of distrust that many residents of the Duwamish Valley have towards academics, as the Duwamish Valley residents have been historically over-studied to fatigue as part of the Superfund cleanup efforts. Instead, the team kept notes on personal achievements for 18 months after project completion, documented the number of hours contributed by Community Scientists, and recorded key demographic factors of participants to examine the six project goals (outlined in Sect. 3.1). To obtain the latter, the Community Science Lead gathered information on age, gender, race, student status, and geographic location of where the community member lived upon recruitment and onboarding. Interested applicants were also invited to share their related lived, work, and/or academic experiences, and their interest in the program. While lower income residents were prioritized in recruitment efforts, the team determined that gathering personal income information was too invasive for this study, and especially difficult for those who work in the informal or gig economies. Instead, the team relied on existing public datasets to identify candidates from overburdened or environmentally unjust communities based on zip codes (e.g. proximity to polluters, low high school graduation, eligibility for state lunch programs etc.).

Over the two years, 115 people participated in Community Science activities: 44 community members participated in construction, field and/or lab measures (61% of the 72 total applicants); 10 participated in virtual or in-person workshops; an additional 53 university students participated in prototype construction seminar courses; 8 participated in the internship program; and 10 participated in the mentorship program (these were either Community Scientists or interns so these numbers did not get double-counted in the overall participant tally). Community scientists logged 203 field hours, 120 lab hours, and 30 h performing remote data analysis. This contribution was significant; when examining academic scientist and Community Science Lead hours, community scientists contributed 38% of total field hours.

Community participants’ ages ranged from 12 to 75, 55% were high school or undergraduate students, 52% were residents from historically marginalized Duwamish Valley and South Seattle neighborhoods, 59% self-identified/expressed female, 70% self-identified/expressed BIPOC (Black, Indigenous, People of Color), 43% self-identified/expressed female BIPOC, and 50% reported no prior scientific or environmental field or lab experience. Two thirds of project interns self-identified/expressed BIPOC, and all mentorship participants were youth of color. Community Scientists were local environmental justice advocates, teachers, STEM after school program organizers, environmental group board members, high school to graduate school students, retirees, and people between employment. The majority stated their interest in the project came from desires to learn about environmental justice histories in the Duwamish Valley, actively contribute to local positive action-oriented research, learn new skills in environmental research, or apply past academic coursework in real-life settings. Of note, many expressed barriers to engaging in these types of opportunities in the past due to volunteer-only expectations, limited transportation means in their household, and/or not knowing someone personally in NBS related fields. Students who participated in project university courses, independent projects, or field and lab activities came from 15 different majors.

### Documentation of public outreach engagement

To gather information on active public outreach, the team tracked the attendance of in-person events. For passive public outreach, the team registered the average number of “people reached” (the number of unique people who saw the content at least once) and “people engaged” (the number of unique people who reacted, commented or shared content) on Facebook postings, the number of “followers” and average number of “likes” on Instagram. Because of the nesting within a larger university website, exact data on traffic to the project website was not able to be acquired.

For active outreach, the estimated total attendance at the in-person events the first year was 305 people. For passive outreach, there were significant differences between the first and second year of the study, likely due to the hiring of two communications specialists and the strengthening of community networks. In the first year, there were 8 Facebook postings with an average of 4 “people reached” per post (highest posting was also 4), and an average of 2 “people engaged” per post (highest posting was 7); there were 11 Instagram postings with an average of 3 “likes” (highest posting was 13). The second year of the study, there were 25 Facebook postings with an average of 94 “people reached” per post (highest posting was 563) and an average of 14 “people engaged” (highest posting was 115); there were 16 Instagram postings with an average of 13 “likes” (highest posting was 40). The project Instagram had 316 “followers” by the end of the project. While it was not feasible in the scope of this study to collect information on individuals or organizations involved in the study that made their own social media postings about the project, the inclusion of the Community Liaison, interns and partner organizations contributed greatly to spreading the word in the second year of the study.

### Long term impacts

The team understands that the value of a community science and outreach program may not be seen for years after the project completion and achievements cannot be solely credited to the program. Following up 18 months after project completion, more than half of youth pursued higher education in social justice or environmental careers, and several led efforts to expand BIPOC involvement in local community-led environmental justice organizations including green stormwater infrastructure and urban food justice initiatives. Several Community Scientists applied for graduate school to address environmental injustice through public policy and administration, landscape architecture or environmental fields. Three educators completed their teaching certifications or returned to teach science in Washington State rural school districts. One of the project interns created a website PhotoVoice project about their reflections from the field season during the global pandemic, which became their first poetry chapbook. Another project intern working on the public outreach communications received fellowships supporting a career in investigative journalism. Eighteen months after the project’s conclusion, Community Scientists requested and received eight letters of recommendation or references for next career moves. One intern who worked in the lab analyzing invertebrates was accepted into a research assistantship in a university medical lab that also utilized invertebrates. One Community Science Lead went on to work for the Duwamish River Community Coalition, the other Community Science Lead is leading environmental justice, restoration and STEAM youth work around the country, and the Community Liaison went on to direct the Duwamish Valley Sustainability Association working with BIPOC and underserved youth, including the recent hire of a former project intern. Public acceptance of floating wetlands in Seattle appears to be growing. The Port of Seattle noted that because of their participation in the Duwamish River Floating Wetlands project, they were able to facilitate partnering with local environmental groups to test other NBS prototypes in other areas around Seattle. Many of the contacts that were initiated with the Duwamish River Floating Wetlands project were strengthened in subsequent projects.

## Discussion

### Reflections on project impact

With dedicated funding, representative leadership, and partnerships with over two dozen community supporting organizations across the public, private, non-profit and academic sectors, the Duwamish Floating Wetlands Project successfully embedded community science and public outreach in each step of the NBS. Comprising over a third of the field hours and engaging 115 people over two years, the contribution of Community Scientists to the project was significant. By prioritizing underserved and local community-based groups, and providing a diversity of opportunities for involvement, the project met the six goals of the project, and the long-term impacts of the project are still being realized. The latter is especially relevant in regards to local capacity building and opportunity for local community members to see science, design, and policy fields in action using NBS to address their environmental injustices and provide ecosystem services to their communities. While this project showcases one way to hold social equity and justice at the forefront of NBS goals, it was not possible to measure the true impact of community participation on enhancing ecological goals.

One of the most significant, yet not systematically documented, outcomes of the project came from meaningful exchanges and reciprocal knowledge between the wide diversity of people and organizations involved in the project. For youth and early career Community Scientists, having personal formal experiences as well as casual field and lab conversations with academic scientists and professionals helped them learn firsthand about higher education, research methods, or career path options. They acquired science skills alongside translatable social, organizational and networking skills which are often necessary to succeed in higher education classrooms and real-world settings (e.g. science vocabulary, discipline in protocols, etc.). Perhaps most importantly, they built memories and a deeper relationship to the river itself, expanding their personal environmental ethics. Community Scientists would often excitedly send Community Science Leads photos of ecologically significant moments unrelated to the project while out in the field (e.g. spotting river otters, or an oil slick). Furthermore, Community Science Leads report that during project orientation several Duwamish Valley participants confessed they were barely aware of the river prior to this project, compared to dialogue at the end of the project, when these same participants were casually discussing ecological and cultural factors of the river. For the academic scientists and professionals, these personal experiences with community members expanded their knowledge base as well. Many Community Scientists had lived knowledge of the Duwamish River and environmental injustices impacting the surrounding neighborhoods. For example, upon noticing a gasoline spill on the river during fieldwork, several Community Scientists used their knowledge from working with local organizations to take charge of the situation. Another Community Scientist pulled from previous experiences conducting salmon research in Alaska and shared suggestions that helped improve upon study methods for fish documentation. Many Community Scientists shared their personal histories living or working on the Duwamish River which helped to provide socio-environmental context to the work. Conversely, other Community Scientists shared they had never been to the Duwamish River, despite living in the adjacent neighborhoods their entire lives—emphasizing the lack of public accessibility in this heavily industrialized section of the river. This information helped shape the public outreach and advocacy strategy for floating wetland systems and river restoration, important to influence voting and funding of future NBS projects.

### Challenges and limitations to inclusivity

The pandemic was a significant limitation in the study. The team had intentions to ramp up the public outreach and community partnerships to be more inclusive towards Indigenous partners, undocumented sustenance fishers, and immigrants or people who spoke a language outside of English. However, this work could not be done without in-person meetings to build trust that were not possible in Spring 2020.

Because of the connections of project leadership, most of the participating students were from a large public university with primarily graduate and four-year undergraduate degrees, albeit many were first in their families to attend college. In future projects there is room for internships to be meaningfully extended to high school and community college students in priority neighborhoods, and non-traditional or reengaged learners referred by partner community-based organizations. Adding more Community Liaisons and multilingual project materials could have helped reach communities not formally associated with organizations or those who speak other languages. A shortcoming of the project was limited connection points with the Indigenous communities, primarily due to the lack of Indigenous connections in project leadership. An Indigenous person as a Community Liaison may help to build trust and reach these communities. Lastly, future studies of NBS could provide more opportunity for the research questions, methods and analysis to be directly influenced and shaped by Community Scientists to increase the meaningful reciprocal exchange of lived and academic knowledge. This might entail community workshops, focus groups, and more time strengthening relationships in the community and in the field.

## Guidelines for future teams

We outlined the frameworks, approach, and outcomes of the community science and public outreach program as part of the Duwamish Floating Wetlands Project and reflected upon the lessons learned in the two years of this study to provide funders, practitioners, researchers and communities insight into the value of prioritizing social equity, environmental justice and community involvement alongside ecosystem needs in NBS. We gathered our lessons learned from different perspectives of team members (Community Science Leads, the Project Investigator and Project Manager, and members of the academic and community teams) and compiled them into a list of suggested guidelines, outlined in Table 1.

**Table 1:** Suggested guidelines for working with community scientists in nature-based solutions

**1. Be educated and focused in your intent.** Prepare a holistic and focused vision and mission statement and share it in a culturally responsive, clear and consistent way to your priority audiences. Be sure to learn local histories and present environmental injustices and conditions before reaching out to community members; do not expect them to teach you, share their pain, or knowledge
**2. Be adaptive and open to community influence.** Allow the vision to reflect local community values and related efforts. Share this vision with individuals and organizations who live and/or work in your target communities. Listen. Be open to refining the project to remain accountable to suggestions, questions, related work underway, gaps identified or strengthened partnerships. If possible, offer tokens of gratitude for this critical early stage feedback (e.g. honorarium, a meal etc.). In the field, encourage reciprocal learning and knowledge exchange between academic and community scientists. Allow the project to adapt to the inclusion of community voices and local knowledge of place and communities
**3. Be realistic in limitations of personal biases.** Formulate the project team, including project leadership, to reflect the diversity of the prioritized audience for the community science program. Consider gender identity, race, income, age, disability, language, education, immigrant status, housing status, proximity to project etc. to maximize success in recruitment, trust-building, stewardship or authentic public support for the project. Equitably budget incomes for all team members to reflect combined lived and professional experiences and offer higher pay for critical skills (e.g. multiple languages, trauma-informed training etc.). Create meaningful partnerships with community groups or organizations where team composition gaps occur, and be genuine and transparent about intentions to partner
**4. Be clear in communications.** Make no assumptions about what the community and academic team understands, and break down terminology, methods, health and safety, and youth engagement protocols into common language and graphics. Many community members do not typically communicate in methods common to universities or scientific communities (e.g. email) and vice versa. Use a diversity of communication tools such as emailed graphic handouts, a group texting platform, personal phone calls and hands-on training workshops to accommodate the needs and comfort styles of all participants. Create project materials in platforms that can be easily translated or made accessible to those with varying learning or physical abilities
**5. Be organized, consistent and transparent to build trust.** Establish a clear and welcoming platform to collect contact information and organize schedules for team, participants, and project partners. Public outreach should be consistent in messaging and harness communication styles of community networks (e.g. social media, fliers on community billboards, culturally reflective language videos etc.). Provide hard copies of all documents to the Community Scientist for their personal records. Be transparent about what you will be doing with the data they collect, photos or findings from the study, or use of their name in credits or promotion; obtain consent. Whenever possible, report back study findings to participants and organizations
**6. Be present and available to build trust.** Provide multiple correspondences and friendly reminders of scheduled field or lab days, required paperwork, or to answer individual questions before scheduled shifts. If possible, join the community member in the field so there is a friendly face in their experience, and support recruiting their peers or family to join the project. Assure all community members have multiple ways of contacting you, and be flexible on when you are available to take calls, texts, etc
**7. Be inclusive.** Create alternative options for interested community members to participate in the project if their schedule or abilities do not match project needs, so you are not turning anyone away. For example, group on-site or virtual workshops for those unable to attend in the field
**8. Be flexible.** If intention is for participation from historically underrepresented groups, allow for flexibility in requirements. For example, many university payment systems require full names, address and social security numbers which may be a barrier to immigrants, refugees, those who live with undocumented families, or those not in stable housing. In addition, the project schedule may need to bend around shifting availability of community members or community events. Communicate this need for flexibility early on to funders and partners so they understand the predictably uncertain nature of meaningful community work
**9. Be gracious.** Working with community members is not just a “nice thing to do”, but rather it can be a critical component to the success, political support and stewardship of the nature-based solution. Support community members in their contribution to science and the project by providing equitable payment, academic credit, a certificate, ongoing mentorship, publication opportunity, or other meaningful output (ask them)
**10. Be accountable.** Be accountable to communities furthest from justice. Acknowledge the places the project is situated within and recognize environmental injustices in the project or context. Own mistakes when they happen and do not offer empty apologies or promises. Open source your publications and consider mutual share agreements of data with Community Scientists and stakeholders
**11. Be patient and acknowledge the long game.** Understand that the value of a community science and outreach program may not be seen for years after the project completion. Ripple effects in personal academic paths, careers, or project momentum take time and often require social capital. If possible, responsibly follow-up with participants. Help keep opportunity doors open for project participants furthest from justice even after the project has winded down
**12. Remember to have fun and show gratitude!** Making place-based science fun increases positive associations and can support breaking down barriers to higher education or educational pathways. Allow for celebrations, tasty treats, music-filled work parties, lively conversations and spontaneous breaks in field activities

## Conclusion

Community Science programs are not only popular, but also play important roles in restoration efforts to gain social acceptance that might influence policy and programs supporting environmental justice and ecological restoration. They also provide an opportunity to work with those groups furthest from justice. This project considers the expanding scope of environmental justice, and the role of training and community participation in addressing equity, environmental literacy and civic activation alongside the socio-ecological impacts of the physical NBS (Chao 2020; Chakraborty 2016). This project was able to successfully involve a wide diversity of community scientists in prototype construction and maintenance, field and lab work, and mentorship and internship programs and workshops, while engaging the general public through both active and passive public outreach. The partnership of professionals, academics, students and community members creates a complex yet rich scientific process that requires significant dedicated time for ethical discussions and meaningful community dialogue. Community contribution to the science of the NBS was significant and personal experiences may have long-term impacts on individuals, environments and community alike. Challenges included project adaptations to the global pandemic, limited involvement with certain groups such as Indigenous peoples and reengaged learners, and limited opportunity for co-development of research methods. Finally, a list of 12 logistical and conceptual guidelines compile the lessons learned from this project to advise future teams looking to develop community science programs in their NBS projects.
